# Septic arthritis of the wrist: about six cases

**DOI:** 10.11604/pamj.2019.33.237.14926

**Published:** 2019-07-19

**Authors:** Mohamed Amine Triki, Nader Naouar, Sofien Benzarti, Hamdi Kaziz, Thabet Mouelhi, Mohamed Laaziz Ben Ayeche

**Affiliations:** 1 Orthopedic Surgery and Trauma Department, Sahloul Hospital, University of Sousse, Sousse, Tunisia

**Keywords:** Septic arthritis, wrist, treatment, surgery, antibiotics, results

## Abstract

The wrist is a rare location of septic arthritis. It often involves patients with preexisting joint disease which symptoms could be confused with infection making the diagnosis more difficult and usually delayed. It is often responsible for residual functional impairment and for a high mortality rate among vulnerable patients. We report 6 cases of septic arthritis of the wrist in 3 males and 3 females. The mean age was 32 years in the male patients and 66 in the female patients. All the women were followed for rheumatoid arthritis. Biological results showed elevated rates of white blood cells and c-reactive protein in all the patients. Joint fluid analyses showed elevated white blood cell count. The treatment was medico-surgical consisting in synovectomy, joint debridement and immobilization of the wrist. At the average follow-up of 1 year and 4 months, 3 patients recovered a perfect mobility of the wrist without any limitation of the range of motion nor the strength. Three patients developed stiffness of the wrist.

## Introduction

The incidence of septic arthritis is stable despite the advances in antibiotic therapy. The involvement of the wrist is rare and represent only 3% of all septic arthritis [1]. It is often responsible for residual functional impairment and for a high mortality rate among vulnerable patients. Risk factors include older age, rheumatoid arthritis, immunodeficiency, diabetes mellitus and a preexisting joint disease to which the symptoms could be confused with infection.

## Methods

We performed a retrospective study of 6 patients treated in our department, during a period of 13 years (January 2005 to December 2017). Epidemiological data, clinical features, investigations, treatment and follow up of the patients with this type of injury were analyzed.

**Patient consent and ethical approval:** written informed consent was obtained from the patients for publication of this Case series and any accompanying images. A copy of the written consent is available for review if necessary. The study was approved by the institutional review board.

## Results

**Figure 1:**
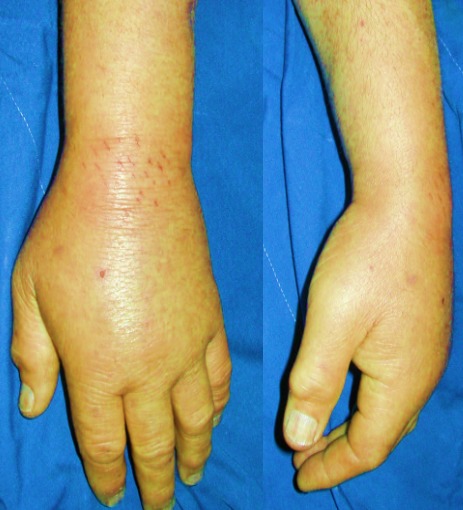
Patient presenting with painful left wrist and fever with sequels of scarification

**Figure 2:**
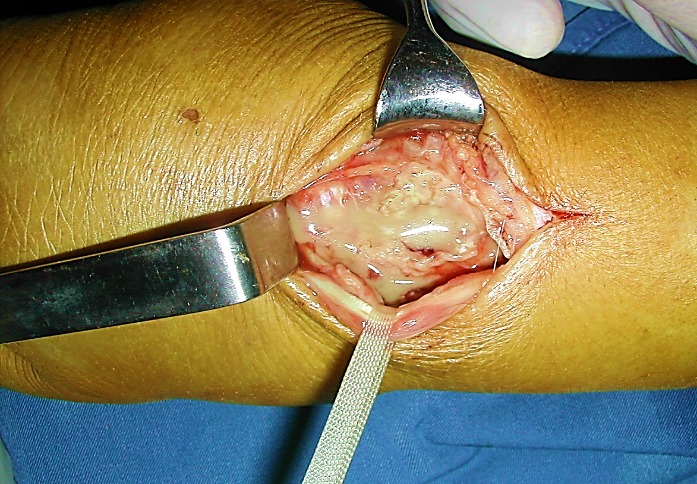
Synovectomy of the wrist and joint debridement

**Figure 3:**
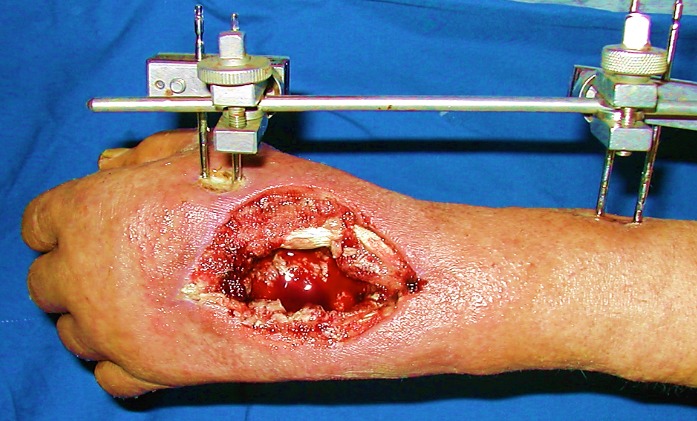
Immobilization with external fixator

We report 6 cases of septic wrist arthritis in 3 males and 3 females. The 3 men were young with a mean age of 32 years and the infection was due to septic inoculation; an insect bite complicated with cellulitis of the wrist and extension to the joint in 1 case and an infection of scaphoid fracture pinning in 2 cases. All the women were followed for rheumatoid arthritis with a mean age of 66 years (Table 1). All the patients presented with painful wrist and fever without a recent history of trauma. One patient had scarifications on the dorsal aspect of the wrist (Figure 1). Biological results showed elevated rates of white blood cells and c-reactive protein in all the patients. Aspiration of the wrist was attempted in all the patients. Joint fluid analyses showed elevated white blood cell count. The treatment was medico-surgical in all cases consisting in synovectomy of the wrist, joint debridement and immobilization of the joint with an external fixator associated with antibiotic therapy during 4 to 6 weeks adapted according to cultures (Figure 2, Figure 3). Bacterial culture was positive in 4 patients (2 staphylococcus aureus, 2 streptococcus A). Histological study of synovial biopsies did not show a specific appearance. At the average follow-up of 1 year and 4 months, 3 patients recovered a perfect mobility of the wrist without any limitation of the range of motion nor the strength. Three patients developed stiffness of the wrist.

**Table 1:** Features of the different cases of our series

Case	Age	Sex	Risk factor	Surgical management	Bacteriology	Post-operative range of motion
1	64	Female	Rhumatoïd Arthritis	Synovectomy + Articular debridement + External fixation	Staphylococcus Aureus	Stiffness
2	68	Female	Rhumatoïd Arthritis		Staphylococcus Aureus	Stiffness
3	66	Female	Rhumatoïd Arthritis		Streptococcus A	Good range of motion
4	34	Male	Scaphoïd Fracture Pinning		Streptococcus A	Stiffness
5	30	Male	Scaphoïd Fracture Pinning		Negative	Good range of motion
6	32	Male	Wrist cellulitis secondary to an insect bite		Negative	Good range of motion

## Discussion

The wrist is a rare location for septic arthritis. It represents only 3% of all septic arthritis [1] and it was involved in only 1.5% of patients presenting specifically for a painful wrist without trauma in a series of 892 patients [2]. A thorough history and physical examination remain critical elements in assessing a suspected wrist infection in a painful wrist without previous trauma [3]. Wrist pain, stiffness, decreased strength of hands and wrists, fever, chills, history of recent or distant infection, direct inoculation of a joint, or recent surgery are common problems [3, 4]. Septic arthritis of the wrist relies heavily on the analysis of joint fluids. Thus, aspiration should be attempted in any patient with a suspected wrist joint infection [5]. Since the same physiopathologic process is responsible for larger joint infections, it can be expected that the confirmatory cell count values observed in these cases would be applicable to articular infections of the wrist [4, 6]. Positive gram staining and culture can both confirm the diagnosis of septic arthritis and guide antibiotic therapy. Although many organisms may be responsible for an infection of the wrist joint, *staphylococcus aureus* remains the most common. In up to 40% of cases of septic arthritis of the wrist, no organism is identified [7]. Surgical management of septic arthritis of the wrist involves open arthrotomy of the radiocarpal joint, with extension into the medio-carpal area as needed. The radiocarpal joint is usually accessible by a standard dorsal approach, which begins with a dorsally medial incision, followed by arthrotomy of the wrist joint between the third and fourth extensor compartments [7]. After culture was obtained, the radiocarpal and midcarpal joints should be washed out with normal saline. If clinically justified, exposure and irrigation of the distal radioulnar joint could be achieved [8]. Antibiotics should be withheld until cultures are obtained, after which a broad-spectrum empirical coverage antibiotic should be started. Recommended empirical intravenous therapy includes vancomycin and another antibiotic that treats methicillin-resistant *Staphylococcus aureus*, then adapted according to definitive laboratory cultures [5].*Staphylococcus aureus* is the most frequently identified organism in cases of septic arthritis of the wrist. *Streptococcus* is the second most common, followed by gram-negative organisms. Generally, intravenous treatment is indicated for 1 to 2 weeks, followed by oral treatment for an additional 2 to 4 weeks [5]. The outcome of septic arthritis of the wrist is usually favorable when treatment is appropriate, using surgical debridement and concomitant antibiotic therapy without delay. In a series of 29 wrists, Rashkoff showed better results when the arthrotomy was performed within 10 hours of diagnosis [8].

## Conclusion

Septic arthritis of the wrist is a diagnostic and therapeutic emergency. The diagnosis of septic arthritis is largely based on anamnesis and physical examination and a high level of suspicion must be maintained. Aspiration of the wrist should be attempted in all cases with high clinical suspicion. Treatment consist in surgical debridement and immobilization of the wrist associated with antibiotic therapy. Outcome is usually favorable but delayed diagnosis can result in permanent joint destruction.

### What is known about this topic

- The wrist is a rare location of septic arthritis;
- Risk factors include older age, rheumatoid arthritis, immunodeficiency, diabetes mellitus;
- Treatment is medico-surgical consisting in synovectomy, joint debridement, immobilization and antibiotics.

### What this study adds

- Aspiration of the wrist should be attempted in all cases with high clinical suspicion.
- Better results are obtained when the arthrotomy is performed within few hours of diagnosis.

## Competing interests

The authors declare no competing interests.
